# From DNA to FBA: How to Build Your Own Genome-Scale Metabolic Model

**DOI:** 10.3389/fmicb.2016.00907

**Published:** 2016-06-17

**Authors:** Daniel A. Cuevas, Janaka Edirisinghe, Chris S. Henry, Ross Overbeek, Taylor G. O’Connell, Robert A. Edwards

**Affiliations:** ^1^Computational Science Research Center, San Diego State University, San DiegoCA, USA; ^2^Mathematics and Computer Science Division, Argonne National Laboratory, ArgonneIL, USA; ^3^Fellowship for Interpretation of Genomes, Burr RidgeIL, USA; ^4^Biological and Medical Informatics Research Center, San Diego State University, San DiegoCA, USA; ^5^Department of Computer Science, San Diego State University, San DiegoCA, USA; ^6^Department of Biology, San Diego State University, San DiegoCA, USA

**Keywords:** metabolic modeling, metabolic reconstruction, *in silico* modeling, flux-balance analysis, model SEED, genome annotation

## Abstract

Microbiological studies are increasingly relying on *in silico* methods to perform exploration and rapid analysis of genomic data, and functional genomics studies are supplemented by the new perspectives that genome-scale metabolic models offer. A mathematical model consisting of a microbe’s entire metabolic map can be rapidly determined from whole-genome sequencing and annotating the genomic material encoded in its DNA. Flux-balance analysis (FBA), a linear programming technique that uses metabolic models to predict the phenotypic responses imposed by environmental elements and factors, is the leading method to simulate and manipulate cellular growth *in silico*. However, the process of creating an accurate model to use in FBA consists of a series of steps involving a multitude of connections between bioinformatics databases, enzyme resources, and metabolic pathways. We present the methodology and procedure to obtain a metabolic model using PyFBA, an extensible Python-based open-source software package aimed to provide a platform where functional annotations are used to build metabolic models (http://linsalrob.github.io/PyFBA). Backed by the Model SEED biochemistry database, PyFBA contains methods to reconstruct a microbe’s metabolic map, run FBA upon different media conditions, and gap-fill its metabolism. The extensibility of PyFBA facilitates novel techniques in creating accurate genome-scale metabolic models.

## Introduction

Since the dawn of genomics, homology-based algorithms and annotation databases have been used to infer meaning from raw sequences (Overbeek et al., 2000, 2003; Aziz et al., 2008), and papers describing microbial genomes have summarized the number of metabolic genes and breakdowns of their potential capacity. However, that information was usually presented *in absentia* the biochemical network that it purports to describe. The metabolic summary of a genome was limited to a few tables of higher metabolic categories. Genome-scale metabolic networks have the potential to completely change our perspective of microbial genomics and of the meaning inferred from a genome sequence (Oberhardt et al., 2011; Plata et al., 2015; Yurkovich and Palsson, 2016). By placing the genome annotation in the context of how the biochemical components of the cell combine to consume substrates, produce energy, and grow, genome-scale models demonstrate the breadth of our understanding of an organism whose genome has been sequenced, while also highlighting the gaps in our knowledge that further study will complete.

Flux-balance analysis (FBA), described elsewhere in this special issue have become the *de facto* standard method for predicting the fluxes through the reactions in the metabolic network, and thereby asserting which biochemical reactions are complete in the organism. FBA is a constraint-based linear optimization approach to solving the flow of compounds through a metabolic network in order to predict cellular phenotypes (Palsson, 2000; Edwards et al., 2002; Orth et al., 2010). The reactions are written as equations, with compounds being converted from substrates to products. A single equation is included in the system that represents the *objective function*, the equation that is targeted to be optimized. In order to get growth (biomass production), ATP production, or any other output of the system, the system of equations representing the cell must produce a solution that results in flux through that single equation that represents the objective function; the optimization is typically to maximize the amount of flux through that equation. In modeling bacterial cells there are almost always more reactions than there are compounds (whose concentrations are unknown) that describe the system. For example, the *Citrobacter* model we recently published recently contains 1,399 reactions (columns) and 1,301 compounds (rows) (Cuevas et al., 2014). Therefore, these models are mathematically underdetermined and the only way to solve them is to apply specific constraints to the system (Kauffman et al., 2003).

The process of running FBA can be broken down into two broad objectives: creating the mathematical model and solving the mathematical model. Solving the mathematical model is straightforward and is usually performed by an optimization library. There are a number of alternatives including the Open Source Gnu Linear Programming Kit (GLPK) (Makhorin, 2008), the commercial (MATLAB, 2012) linprog^1^, and IBM ILOG CPLEX Optimization Studio (IBM ILOG, 2014) is not the focus of this work. Creating the mathematical model is much more complex, as it requires incorporating biological knowledge to transition between DNA sequence, functional roles, enzymes, and reactions. Including other metabolic-related sources of information has also been used to build these models (Lee et al., 2006; Raman and Chandra, 2009; Carrera et al., 2014; Liu et al., 2014). There are several software packages designed to do some or all of these steps for you, such as the COBRA Toolbox (Schellenberger et al., 2011; Ebrahim et al., 2013), KBase (Overbeek et al., 2013), the Systems Biology Research Tool (Wright and Wagner, 2008), FASIMU (Hoppe et al., 2011), CellNetAnalyzer (Klamt and von Kamp, 2011), the Model SEED (DeJongh et al., 2007; Devoid et al., 2013), and others (Lakshmanan et al., 2012; Hamilton and Reed, 2014).

In this paper we describe the process of generating a metabolic reconstruction and running FBA starting with a genome sequence. We demonstrate how to identify the reactions present in a model derived from a genome, and how to convert those reactions to a stoichiometric matrix. We demonstrate how to identify additional reactions that need to be included in the model, and reactions that can be excluded, and how to test the model under different growth conditions. We introduce a new open source library, PyFBA, that allows bioinformaticians to build and explore FBA models using the Python programming language and that is freely available to all researchers. We explain each of the steps required to go from DNA to FBA for the bioinformatician.

## From DNA to FBA

The steps from DNA to FBA include identifying the functional roles in the genome; connecting those roles to enzyme complexes and then to reactions; converting those reactions to equations that describe the conversion of substrates to products; defining the growth media and external conditions; and testing growth of that model. Usually, developing a complete metabolic model requires several iterations of adding reactions to enable the model to grow and removing reactions to limit the growth of the model under conditions where it should not grow. We discuss each of these steps individually below.

### PyFBA

We have developed a Python code base, PyFBA, that allows you to build a genome-scale metabolic model and run FBA on that model. The PyFBA code is available from GitHub or the Python Package Index repository under the MIT License (Cuevas et al., 2016a,b). PyFBA works with the GNU Linear Programming Kit (GLPK) or the IBM ILOG CPLEX Optimization Studio for solving the linear system. In the examples below we use this code to demonstrate how to go from DNA to FBA. To install the PyFBA code, see the detailed instructions available online at http://linsalrob.github.io/PyFBA/installation.html.

### Genome Annotations

The first step in building a metabolic model of an organism is to identify all the genes present in that organism. There are a number of tools for genome annotation, including RAST (Aziz et al., 2008; Overbeek et al., 2013), PROKKA (Seemann, 2014), BG7 (Tobes et al., 2015), Blast2GO (Conesa et al., 2005), and BASys (Van Domselaar et al., 2005). Most of these tools take unannotated contigs, and iterate through steps for accurately identifying the protein- and RNA-encoding genes and assigning functional roles to those genes. Although all of these tools will identify most of the metabolic genes in the genome and will provide accurate annotations of those genes [including Enzyme Commission (EC) numbers, see below], connecting those annotations to enzymes and then to reactions is a complex undertaking for the output from most tools. In this paper we use annotations generated by RAST to connect to biochemical reactions encoded by the Model SEED as both the functional roles and connections to enzymes are publicly available and frequently updated. PyFBA does not require RAST annotations, but does require a connection from annotation to biochemistry.

The list of functional roles in a genome can be downloaded from the RAST website in several different formats: for example, from the *Job Details* page of an annotated genome, the annotations can be downloaded as spreadsheets (the easiest to use in model building), GenBank files, GFF files, or RAST genome directories.

### Converting Functional Roles to Reactions

After identifying the protein encoding genes present in the organism, and assigning functions to those proteins, the enzyme complexes that are created by those proteins must be characterized. EC numbers (Webb, 1992) are most often used when making these mappings between different repositories because they are the most widely applied annotation to gene products. However, EC numbers do not cover all reactions in microbial cells, and with different annotation naming conventions and nomenclature, automated processes to compile the list of reactions is extremely difficult. However, using a single database (e.g., the SEED that underlies the RAST platform) provides a convenient connection between functions, enzyme complexes, reactions, and compounds because of the consistent work of the annotators.

**FIGURE 1:**
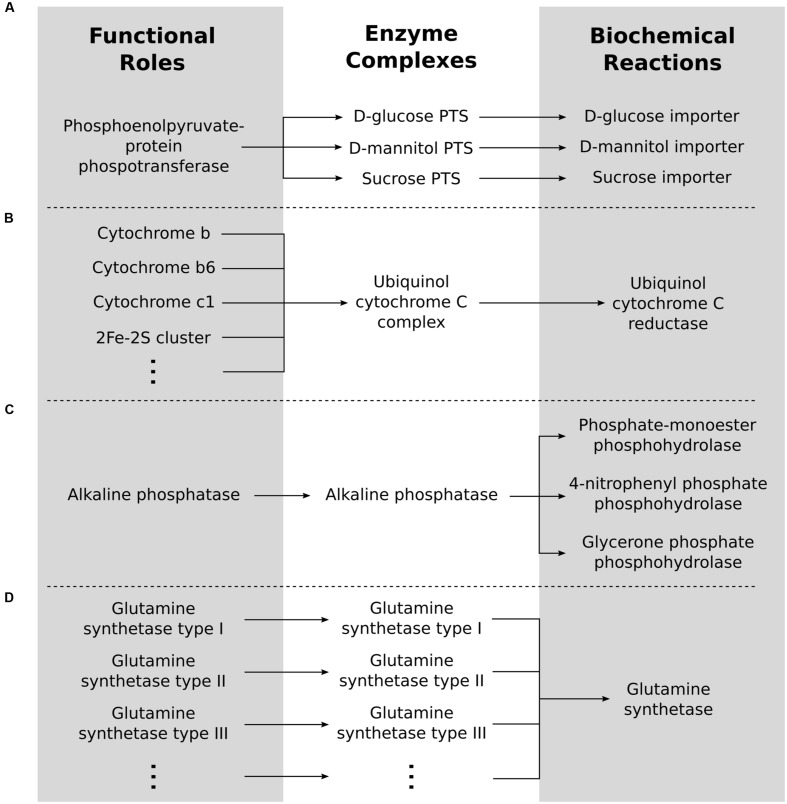
**Roles to complexes to reactions.** Functional roles have a many-to-many relationship with enzyme complexes. Similarly, enzyme complexes have a many-to-many relationship with biochemical reactions. **(A)** A one-to-many relationship from roles to complexes. **(B)** A many-to-one relationship from roles to complexes. **(C)** A one-to-many relationship from complexes to reactions. **(D)** A many-to-one relationship from complexes to reactions.

Enzyme complexes can be formed by one or several functional roles, and each functional role can be involved in one or more complexes, illustrating a many-to-many relationship (**Figure 1**). For example, the functional role “Phosphoenolpyruvate-protein phosphotransferase of PTS system (EC 2.7.3.9)” encoded by the *ptsI* gene in *Escherichia coli* is involved in several different complexes each associated with the import of a different sugar (**Figure 1A**); the Ubiquinol-cytochrome C complex requires ten different functional roles each encoded by a separate gene (**Figure 1B**); and the role “Alkaline phosphatase (EC 3.1.3.1)” encoded by the *phoA* gene in *E. coli* is in a complex by itself (**Figure 1C**). The first step in identifying the reactions that are encoded by a genome is therefore to convert the functional roles associated with the proteins identified in the genome to enzyme complexes that are functional in the cell. Comparably, each reaction in a cell can require one or more complexes, while each complex can be involved in one or more reactions, thus creating another many-to-many relationship. For example, the complex created by alkaline phosphatase is responsible for many dephosphorylation reactions (**Figure 1C**) while the dodecomeric glutamine synthetase catalyzes a single reaction (**Figure 1D**).

To convert functional roles to reactions roles must first be connected to enzyme complexes. If the annotation system used to identify the roles in the genome only provides EC numbers, these need to be connected to complexes. Most subunits of the same enzyme complex are given the same EC number; for example all the subunits of ATP synthase are given the EC number 3.6.3.14, which facilitates joining reactions into complexes. The connections between functional roles (and EC numbers in particular) and reactions or pathways can be obtained from several public resources, such as EXPASY^2^ (Gasteiger et al., 2003), the KEGG REACTION database^3^ (Kanehisa et al., 2004), MetaCyc^4^ (Caspi et al., 2014), and BRENDA^5^ (Schomburg et al., 2002). As an alternative, the Model SEED^6^ (Overbeek et al., 2013) maintains a mapping between functional roles and complex IDs and a separate mapping between complex IDs and reactions.

### Converting Reactions to a Stoichiometric Matrix

**FIGURE 2:**
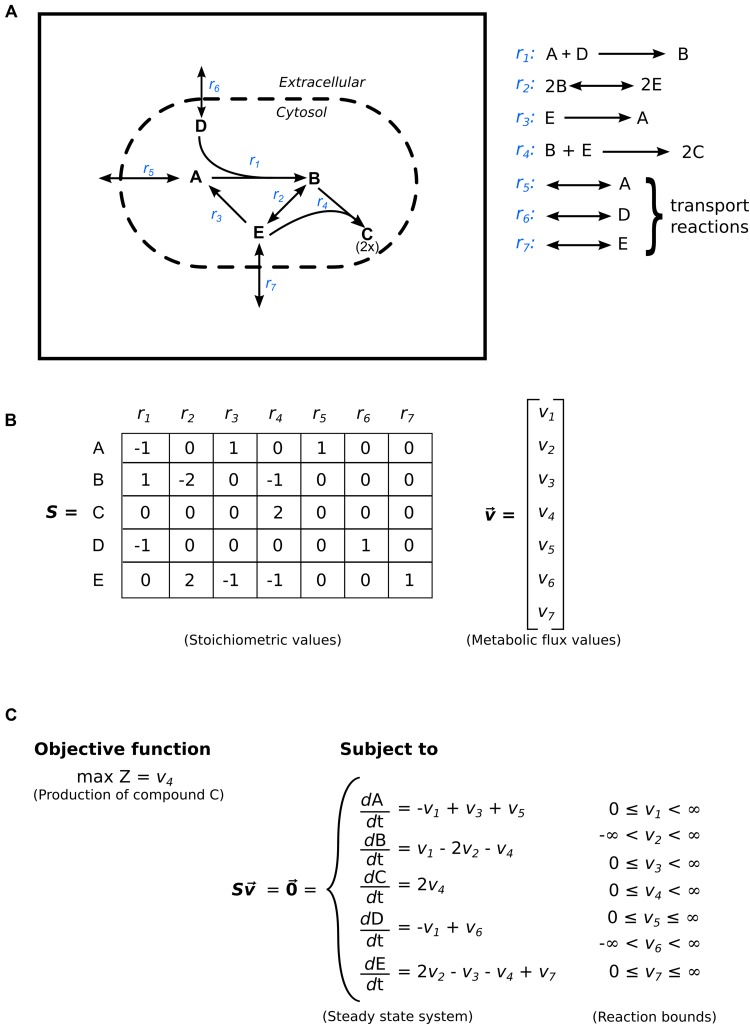
**Flux-balance analysis (FBA).** **(A)** Example of a bacterial metabolic model displaying two compartments separated by a dashed boundary (extracellular and cytoplasm), seven reactions labeled in blue text (four intracellular and three transporters), and five compounds. **(B)** The stoichiometric matrix ***S*** with corresponding stoichiometric coefficients, and the flux vector ***v.*** Each matrix-cell represents the number of compound molecules required for the particular reaction. The integer sign denotes the compound as a reactant (negative value) or as a product (positive value). A zero means the compound is not involved in the reaction. Reversible reactions are typically present in the matrix in one direction. In the instance that a reaction is reversed in the solution, the metabolic flux value for the corresponding reaction will be negative, thus indicating a switch in directionality. **(C)** The linear programming problem. The mass balance equations constrain the change of compound concentration over time to zero. The final constraints on the system are the physicochemical enzymatic bounds. These bounds signify directionality (e.g., *v*_3_ has a lower bound of 0 and can only produce compound A, whereas *v*_2_ is completely unbounded and can proceed in both directions). The objective function is indicated to maximize the flux of *v*_4_ (i.e., the production of compound **C**).

A genome-scale metabolic model starts with a list of reaction equations, compounds, and compartments, and for the mathematical solution we convert that to a *stoichiometric matrix*, essentially a table of reactions and compounds (**Figures 2A,B**). The stoichiometric matrix provides the first level of constraint on the metabolic system — it contains only those reactions and their associated metabolites present within the network, defining the feasible space of phenotypes the system can express. The cells of the matrix represent the relationship between each of the compounds and each of the reactions in the network; thus, a reaction that is not included in the stoichiometric matrix is not included in the model. All phenotypes recognized in the cell must be included in the stoichiometric matrix for an accurate metabolic model.

To construct the stoichiometric matrix, all of the compounds used in all the reactions are stored in the rows of a matrix. All the reactions used in the model are stored in the columns of the matrix, and the individual cells contain the stoichiometry of each compound in each reaction, with negative values indicating that the compound is consumed in the reaction; zero indicating that the compound is not involved in the reaction; and positive values indicating that the compound is produced by the reaction. Most of the values in the stoichiometric matrix are zero because most of the compounds are not involved in many reactions. The dimensions of the matrix are the number of compounds and the number of reactions; thus for the *Citrobacter* model discussed earlier (Cuevas et al., 2014) the matrix was 1,301 × 1,399, providing 1,820,099 possible combinations of reactions and compounds, but only 6,355 values (0.35%) in the matrix are nonzero. **Table 1** illustrates a stoichiometric matrix for the glycolysis pathway (9 reactions, 18 compounds) included in the *Citrobacter* model. The cells that contain a zero have been left blank for visual purposes.

**Table 1:** Glycolysis portion of stoichiometric matrix.

Reaction ID/Compound	Rxn 00216	00558	00545	00786	00781	01100	01106	00459	00148
ATP	-1		-1			1			1
ADP	1		1			-1			-1
D-Glucose	-1								
D-Glucose-6-Phosphate	1	-1							
D-Fructose-6-Phopshate		1	-1						
D-Fructose-1,6-Biphosphate			1	-1					
Glyceraldehyde-3-Phosphate				1	-1				
Glycerone-phosphate				1					
NAD					-1				
Phosphate					-1				
1,3-Bisphospho-D-Glycerate					1	-1			
NADH					1				
H+						1			
3-Phosphogylcerate						1	-1		
2-Phospho-D-Gylcerate							1	-1	
H2O								1	
Phosphoenolpyruvate								1	-1
Pyruvate									1


Compounds in the stoichiometric matrix are also denoted by the compartment in which they are located to differentiate which compounds are required inside the cell from those required outside the cell. The location also provides a convenient mechanism to constrain the model based on which compounds are in the media and which can be transported (see below). Bacterial models typically use just two compartments: intra- or extra-cellular, while models of Eukaryotes often include other compartments, such as the mitochondria or chloroplast (Seaver et al., 2012). For the purpose of these models the Gram negative periplasmic space and things anchored to the outer cell wall are typically considered extracellular. For example, the Gram negative *Citrobacter* model includes 175 compounds in the extracellular compartment.

Preparing the stoichiometric matrix requires iterating through all the reactions and all the compounds associated with those reactions, and creating a table. If the compounds are consumed the coefficient of that compound is negated in the stoichiometric matrix cell. If the reaction is reversed, the coefficients of the appropriate compounds may be negated (although this can also be controlled by the reaction bounds, described below). An example stoichiometric matrix is shown in **Figure 2** and provided in the supplementary table as a tab-separated text file (Supplementary Material 1) that can be imported into any office suite of programs.

### Media Formulation

The media in which the organism is growing defines another constraint imposed upon the metabolic model. Encoding defined media is straightforward, and recipes for almost all media [including complex media such as lysogeny broth (LB) media] are available online. The only difficulty is to ensure correlation between the compound names used in the media formulation and the compound names used in the metabolic reactions. One approach to overcome that obstacle, used, for example, by the Model SEED, is to use a separate database of compounds with unique IDs. By default, of course, the media components have an extracellular location, and the presence of transporters is required to move them into the cell; another constraint imposed on the genome-scale metabolic model. Historically, identification of transporter proteins is challenging because they are largely homologous to each other and only differ by their substrate specificity (Marger and Saier, 1993; Saier, 1994). Latitude is therefore often given in the assertion of which transporter pathways a cell actually has, especially for some of the less well characterized biochemical compounds. Small molecules and ions may diffuse into and out of the cell as well as being actively transported across the membrane, and protein-free reactions invoking those diffusions are included in the cellular model.

### Uptake and Secretion from the Media

The linear programming solution to the genome-scale metabolic model requires that compounds are taken up from the outside (i.e., the media components), and also that some compounds are secreted from the cell (waste products). Moreover, each compound in the stoichiometric matrix must be balanced for a solution to the problem. Therefore, a set of unconstrained reactions are generated that consume all external compounds in the model, but do not do anything with those compounds (in essence, they disappear from the equation; in practice this is akin to them being diluted to extinction in the growth media). These reactions also provide the media compounds as if from nowhere (in practice this is akin to the media compounds diffusing toward the cell as they are consumed). These reactions are sometimes called drain flux reactions or external reactions, and in PyFBA they are called uptake and secretion reactions. Since the set of uptake and secretion reactions is dependent on the external compounds produced by the model and the external compounds present in the media, it is generally calculated for each model upon creation.

### Reaction Bounds

In solving linear equations of underdetermined systems, a defined solution space has to be provided that limits the parameters applied to each of the reactions. It is highly unlikely that one, or a few, reactions would have extremely high fluxes through them while the other reactions in the cell have moderate or low fluxes. Therefore, each reaction in the stoichiometric matrix is controlled by a set of *reaction bounds* that limit the flux through that reaction. In most cases, the reaction bound also assigns the directionality of the reaction. As shown in **Figure 2A**, the direction of reaction *r1* is set from left to right. This is controlled by setting the lower bound of reaction *r_1_* to zero (**Figure 2C**), thus preventing reaction *r_1_* from producing compounds A and D by the linear solver. Furthermore, an alternative approach to designating the directionality of a reaction is to limit the flux through the reaction by negating the coefficients in the equations. The limits are conventionally positive if a reaction proceeds from left to right; negative if a reaction proceeds from right to left; and positive and negative if a reaction is bidirectional. The reactions bounds are applied to the linear solver before the stoichiometric matrix is solved.

**FIGURE 3:**
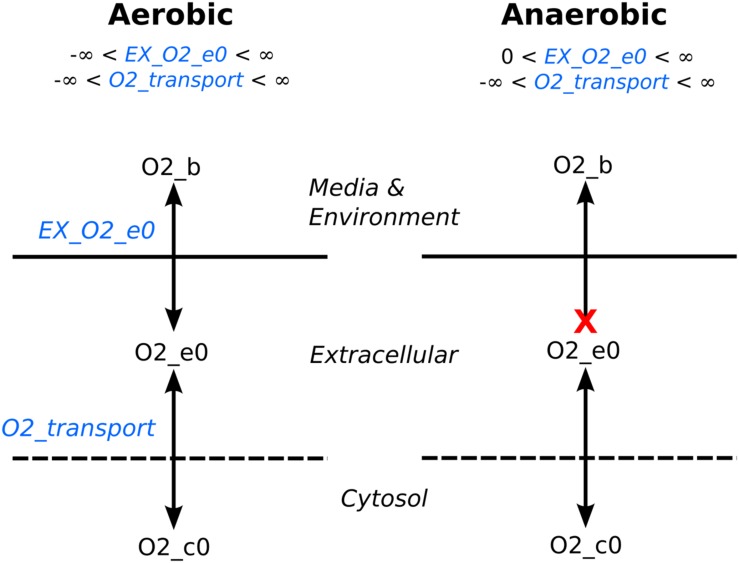
**Controlling oxygen exchange example.** The lower bound in exchange reactions (e.g., EX_O2_e0) moderate the availability of a compound for the extracellular compartment. To simulate an anaerobic environment, flux from the environment to extracellular space is cutoff by setting the lower bound to zero, whereas the lower bound is set to negative infinity for an aerobic environment, allowing unlimited amount of oxygen into the extracellular space.

The media formulation (described above) is typically encoded as a list of compounds that are present in the media, and this is used to determine the reaction bounds of the reactions that transport these compounds into the cell. As we noted, there are a series of uptake and secretion reactions that mimic the diffusion of a media compound toward the cell, and the diffusion of a waste product away from the cell. By setting the bounds of these reactions appropriately, we can control the growth conditions of the cell. If the reaction bounds for diffusion of media components toward the cell are set to allow production of those components and if they are required for growth, they can be consumed. Similarly, the reaction bounds are set to only allow consumption of waste products so that they are removed from the media at the same rate that they are created. The reaction bounds can be manipulated to mimic gene knockouts and environmental changes. For example, simulating an anaerobic environment is accomplished by removing the model’s input oxygen capability by setting the bound to zero (**Figure 3**). The benefit in this method instead of completely removing the uptake/secretion reaction from the stoichiometric matrix is oxygen produced by intracellular reactions could still be secreted from the cell while still preventing oxygen uptake from the environment.

### Compound Bounds

A central tenet of FBA is the assumption that the change in each compound concentration is equal or balanced (**Figure 1C**), and thus overall (with the exception of compounds that are taken up or secreted) the concentration of the compounds sums to 0. This is the steady state mass balance that allows FBA to perform without any kinetic information, and also acts as another constraint on the system. To ensure that the compound consumption is equal to production, the compound bounds are set so that the concentration of the compound can not be greater or less than zero.

### The Objective Function (What We Are Trying to Maximize)

A single reaction is designated as the objective function and is included in the stoichiometric matrix. This is usually the biomass reaction (Supplementary Figure 1), a complex reaction that consists of biomass precursors (e.g., amino acids, nucleotides, carbohydrates, and lipids; but is almost always dominated by the consumption of ATP) and in return produces *biomass*, a generic term to mean growth of the cell. However, the objective function can consume and produce other cellular components. Likewise, typically the solution required maximizes the flux through the objective function (i.e., produce as much biomass as possible), however, with different outcomes represented by alternate equations it may be more suitable to minimize the flux through that equation. The biomass equation reaction is typically added as the first or last row in the stoichiometric matrix.

### Solving the Linear Programming Puzzle

Once the stoichiometric matrix has been constructed, the media defined, the compound and reaction bounds set, and the biomass equation (or other objective function) added to the stoichiometric matrix, the system can be solved by linear programming. The solvers return the value of the flux through the reaction designated as the objective function, with a value above one generally indicating growth, and a value approaching zero indicating no growth (because of the limitations of floating point arithmetic, no growth is not often exactly zero).

### Gap-filling

Typically, a genome-scale metabolic model constructed from the annotations in a genome will not result in growth because the metabolic network is incomplete. Additional reactions have to be added to the model to ensure growth, a process called *gap-filling*. The gap-filling step of building the model is the point where most of the erroneous assertions about the metabolism of an organism are made. A selection of reactions has to be added to the metabolic network derived from the genome annotations, and the selection is often made with little supporting evidence. Consequently, there are many different approaches for identifying the reactions missing from a model, including identifying missing reactions from expression information (Kharchenko et al., 2004), finding reactions that complete the metabolic networks based on their topology (Satish Kumar et al., 2007), using phenotypic data to identify growth conditions (Herrgård et al., 2006; Satish Kumar and Maranas, 2009; Vitkin and Shlomi, 2012; Cuevas et al., 2014), and most recently likelihood based approaches that use sequence similarity (Benedict et al., 2014). Enough reactions could be added that result in the growth of any model on any media, but to retain biological accuracy, only those reactions that result in growth on media where the organism is known to grow should be added to the model.

The gap-filling approach taken by most applications is an iterative approach. Starting with the metabolic model derived from the annotations of the genome, the model is tested for growth. While the model does not grow, additional reactions are proposed, based on a variety of criteria, and the model including the additional reactions are tested again. Once the model grows, gap-generation (see below) can be used to prune unnecessary reactions in the model.

PyFBA includes several different modules for gap-filling, sequentially suggesting reactions to add to the model. For example, there is a set of 109 predefined reactions that are present in every model tested to date and that can be added to gap-fill any model. If an organism is known to grow on a particular media, reactions are also identified to be added to the model that transport the media components into the cell. As noted elsewhere, the identification of transport reactions from genome sequences is problematic, and therefore using phenotype data ensures accurate representation of the biology of the organism in the model. Reactions are identified that connect to orphan compounds – compounds that are only associated with a single reaction in the network. If the orphan compounds are consumed they either need to be produced by another reaction or transported into the cell by a transport reaction. If the orphan compounds are produced in the model they either need to be secreted as waste (via a transport reaction) or consumed in another reaction. A gap-filling approach is also included in PyFBA that analyzes the presence of all the subsystems in the reaction network, and proposes reactions that complete the subsystems in the model. A general framework is also available that accepts tuples of annotations from other genome(s) and the probability that those annotations are associated with the current model and proposes reactions based on those annotations. For example, when building a model of *Citrobacter* a gap-filling approach may be to identify all functional roles annotated in all other *Citrobacter* genomes and the likelihood that the functional role should also be in this *Citrobacter* genome (we typically define this as the fraction of genomes tested that contain this role). Reactions can be suggested for addition to the model based on those annotations and their likelihoods. In addition to suggesting new reactions to the model, the PyFBA gap-filling approach is extensible with new approaches as they are developed.

There are many different approaches that can be used to suggest reactions to be added to models, but most approaches will suggest many more reactions to add than are actually needed for growth. Therefore, excess reactions, that are not absolutely essential for growth, should be trimmed from those suggestions. PyFBA includes a recursive algorithm to reduce a set of proposed reactions from the initial proposal to just those reactions that are absolutely required for growth. Under the best conditions, this algorithm provides *O*(log n) complexity (where n is the number of reactions that are proposed to be added) as it is based on binary search, however, under the worst conditions the algorithm will approach *O*(n) complexity as all the reactions in the model have to be tested.

### Gap-generation

Once a model has been generated that grows under a set of conditions, reactions can be recursively removed until the minimum set of reactions that are required for the model to grow are identified. This approach has *O*(n) complexity (where n is the number of reactions in the model) as each reaction is tested one at a time. It is also worth noting that another method previously developed to identify network redundancy and insubstantial reactions, but is not yet implemented in PyFBA, is flux-variability analysis (Burgard et al., 2001; Gudmundsson and Thiele, 2010). Flux-variability analysis is a linear programming problem that minimizes and maximizes the flux through each reaction while maintaining the same phenotype. Typically, the maximal rate of biomass production is kept constant during flux variable analysis in order to test the flexibility of the metabolic network, or to find ineffective reactions.

### Exchanging Models

Several exchangeable file formats exist that can be used to store metabolic models. The most common among existing tools is the systems biology markup language, or SBML (Lakshmanan et al., 2012). JavaScript Object Notation (JSON) is a widespread key-value format used in many web-based software applications. Consequently, JSON libraries are easily accessible in most programming languages. Both formats are supplied as Supplementary Materials 2 and 3 and are supported by the FBA scripts used here.

## Constructing Genome Scale Metabolic Models Using PyFBA

We have described the principles of how genome-scale metabolic models are created from genome annotations, and in this section we demonstrate how a genome scale metabolic model can be created from genome annotations using PyFBA.

Starting with a genome annotated in RAST, download the annotations as a spreadsheet from the RAST job overview page. This provides a file that can be opened in any office suite of software and contains information about all of the genes identified in the genome, including the location of the protein encoding gene (contig, start, stop, and strand), and most importantly, the function of the protein. We connect the functional roles in the *function* column of this table to the reaction IDs in the Model SEED by using the PyFBA.filters.roles_to_reactions function. This function maps from functional role to reaction ID. We then read an appropriate media file using PyFBA.parse.read_media_file, and either define a new biomass reaction or import one of the predefined reactions from PyFBA.metabolism.biomass.biomass_equation. The stoichiometric matrix is created from these data inputs via the PyFBA.fba.create_stoichiometric_matrix method. The reaction bounds are computed from all the reactions, including the uptake and secretion reactions and the compound bounds (all zero) are added using the two methods PyFBA.fba.reaction_bounds and PyFBA.fba.compound_bounds. Finally, the stoichiometric matrix is solved using the linear programming solver.

**FIGURE 4:**
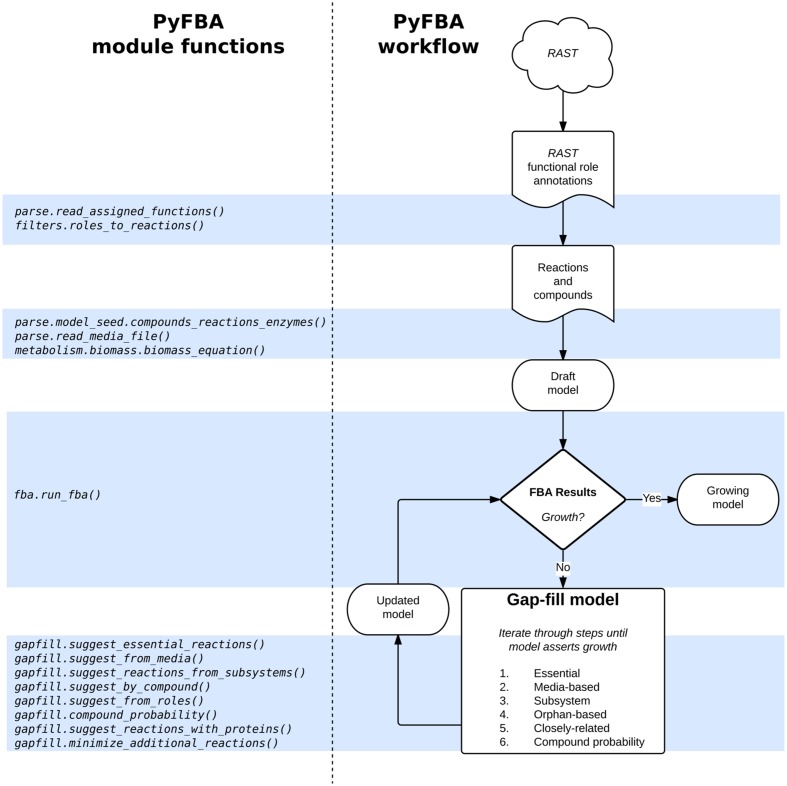
**PyFBA workflow.** Each step to obtain a genome-scale metabolic model using PyFBA is presented on the right. Arrows represent actions to be taken to obtain the next set of information in the workflow. Corresponding to each step are the necessary PyFBA module functions on the left. The gap-fill step is associated with multiple functions, each one differing by the gap-fill strategy used to identify reactions. The gap-fill method is iterative, hence a subset or all of these modules are used to obtain a growing model. The RAST portion of the workflow is an example for using functional role annotations; these steps can be replaced by other methods yielding the same information.

Gap-filling is available in PyFBA using the PyFBA.gapfill modules. As described above, these modules provide several different mechanisms for gap-filling as well as methods to bisect the reactions proposed to be added to your model for growth. **Figure 4** illustrates the typical workflow for PyFBA alongside the key module functions used at each step.

We have created an iPython Notebook^7^ (Supplementary Material 4) that demonstrates each of the steps in creating a model and gap-filling the model using the example data from the *Citrobacter* model discussed previously (Cuevas et al., 2014). A second iPython Notebook ^8^ (Supplementary Material 5) demonstrates how PyFBA can be used to import metabolic models from an SBML document to run FBA.

## Discussion

Genome-scale metabolic modeling extends the usefulness of the base microbial genome annotation by identifying the biochemistry that is actually happening in the cell. The conversion of functional roles to reactions highlights our understanding of the cellular metabolism, while the gaps that remain and need filling before the model “grows” highlight areas where we have less detailed understanding of the biochemistry. PyFBA is a novel Python based implementation of flux balance analysis that can be easily extended and modified to provide new metabolic modeling capabilities.

Gap-filling chooses reactions to add to the model in order to generate growth, and with a database of over 30,000 reactions from which to choose there are many solutions that could result in growth of the model. Therefore, in order to be meaningful, FBA approaches must be constrained using real biological and physicochemical constraints determined from experimentation. Whole genome sequencing and bioinformatics analyses rapidly identify metabolic genes for building draft genome-scale metabolic models (Edwards and Palsson, 2000; Price et al., 2003; Cuevas et al., 2014). Genome-scale databases are expanding and improving, providing more accurate constraints to metabolic models (Overbeek et al., 2013; Wattam et al., 2014; Brettin et al., 2015). More enzymes are being characterized, thus linking new genes to metabolic functions (Kanehisa et al., 2012; Caspi et al., 2014; Sanchez et al., 2015). Accordingly, this will result in more accurate draft metabolic models but will also provide more accurate choices when gap-filling. Transporter reactions are more difficult to differentiate from each other, and some spontaneous reactions do not require any proteins for catalysis. Phenotypic data such as those from minimal media growth experiments provide further evidence to incorporate reactions from particular transporters and enzymes into the metabolic model. Overtime, rendering metabolic models using annotations can be directly improved by the accumulation of unique genomic and phenomic data.

Identifying reactions during gap-filling inadvertently leads to an opportunity to improve genomic annotations. Regions where DNA sequences were not assigned a function due to types of problems with the annotation step, such as the sequence lacking known homology to another organism, can be connected to the gap-filled reactions from PyFBA. This positive feedback has not been efficiently captured by previous software but could provide current databases a means for improving their annotations, narrowing the search space of functional roles the genetic material may be associated with. Future endeavors in model reconciliation will support associations between gap-filled reactions and unannotated genome content.

There are several limitations to the previous versions of genome-scale metabolic modeling that the new iterations of these models overcome. For example, most models lack an integration of regulatory processes in the networks — if a gene is present it is assumed to be expressed and functional. Recent models include transcription factors and gene expression information alongside traditional models (Chandrasekaran and Price, 2010; O’Brien et al., 2013). Another limitation with existing models is the choice of the objective function and the resulting flux distribution through the network. However, the assumption that the flux of compounds through the metabolic networks to optimize growth rate has been shown to correlate with experimental observations (Varma and Palsson, 1993), and maximizing the biomass reaction is consistent with experimental flux data and biomass precursors are in agreement with the experimental observations (Burgard and Maranas, 2003).

## Author Contributions

DC worked on PyFBA development, algorithm design, prepared and wrote manuscript, created figures, and data analysis. JE provided SEED biochemistry resources and discussion. CH provided SEED resources and discussion. RO provided RAST consultation. TO provided algorithm design discussion. RE worked on PyFBA development, algorithm design, wrote manuscript, data analysis, code testing, and code distribution.

## Conflict of Interest Statement

The authors declare that the research was conducted in the absence of any commercial or financial relationships that could be construed as a potential conflict of interest.

## Footnotes

1 http://www.mathworks.com/help/optim/ug/linprog.html

2 http://www.expasy.org/

3 http://www.genome.jp/kegg/pathway.html

4 http://metacyc.org/

5 http://www.brenda-enzymes.info/

6 http://www.theseed.org/models/

7 https://github.com/linsalrob/PyFBA/blob/working/iPythonNotebooks/From_functional_roles_to_gap-filling.ipynb

8 https://github.com/linsalrob/PyFBA/blob/working/iPythonNotebooks/Using_an_SBML_model.ipynb

## Supplementary Material

**FIGURE S1 | Gram-negative biomass reaction.** Coefficients for the reaction are listed before the compound name.

**SUPPLEMENTARY MATERIAL 1 | Sample stoichiometric matrix (*Citrobacter sedlakii*).** *SM1_stoichiometric_matrix.tsv.*

**SUPPLEMENTARY MATERIAL 2 |*Citrobacter sedlakii* SBML file.** *SM2_Citrobacter_sedlakii.sbml.*

**SUPPLEMENTARY MATERIAL 3 |*Citrobacter sedlakii* JSON file.** *SM3_Citrobacter_sedlakii.json.*

**SUPPLEMENTARY MATERIAL 4 | iPython Notebook demonstration of PyFBA workflow for converting functional roles to reactions to gap-filling.** *SM4_From_functional_roles_to_gap-filling.pdf.*

**SUPPLEMENTARY MATERIAL 5 | iPython Notebook demonstration of PyFBA workflow for importing an SBML file and running FBA.** *SM5_Using_an_SBML-model.pdf.*
