# Supplementary material for: From DNA to FBA: How to Build Your Own Genome-Scale Metabolic Model
Source: Front Microbiol. 2016 Jun 17;7:907. doi: 10.3389/fmicb.2016.00907 (PMC4911401; doi:10.3389/fmicb.2016.00907)
Supplement: Supplementary file 5 [file Data_Sheet_4.PDF]

Supplemental Material
PyFBA

From functional roles to gap-filling

Daniel A. Cuevas *Robert A. Edwards*
San Diego State University

1 How to gap-fill a genome scale metabolic model

1.1 Getting started

1.1.1 Installing libraries

Before you start, you will need to install a couple of libraries:

The ModelSeedDatabase has all the biochemistry we'll need. You can install that with `git clone`.

The PyFBA library has detailed installation instructions. Don't be scared, its mostly just `pip install`.

(Optional) Also, get the SEED Servers as you can get a lot of information from them. You can install the git python repo from github. Make sure that the SEED_Servers_Python is in your PYTHONPATH.

We start with importing some modules that we are going to use.

We import *sys* so that we can use standard out and standard error if we have some error messages. We import *copy* so that we can make a deep copy of data structures for later comparisons. We import *print_function* so that we can use the print using Python 3.X syntax. Then we import the *PyFBA* module to get started.

```
In [1]: import sys
        import copy
        import PyFBA
        from __future__ import print_function
```

1.2 Running a basic model

The SBML model is great if you have built a model elsewhere, but what if you want to build a model from a genome?

We typically start with an *assigned_functions* file from RAST. The easiest way to find that is in the RAST directory by choosing `Genome Directory` from the `Downloads` menu on the job details page.

For this example, here is an *assigned_functions* file from our *Citrobacter* model that you can download to the same directory as this iPython notebook. Notice that it has two columns: the first column is the protein ID (using SEED standard IDs that start with `fig|`, and then have the taxonomy ID and version number of the genome, and then `peg` to indicate *protein encoding gene*, `rna` to indicate *RNA*, `crispr_spacer` to indicate *crispr spacers* or other acronym, followed by the feature number. After the tab is the *functional role* of that feature. Download that file to use in this test.

We start by converting this *assigned_functions* file to a list of reactions.

```
In [2]: assigned_functions = PyFBA.parse.read_assigned_functions("citrobacter.assigned_functions")
        roles = set([i[0] for i in [list(j) for j in assigned_functions.values()]])
        print("There are {} unique roles in this genome".format(len(roles)))
```

```
There are 3594 unique roles in this genome
```

Convert those roles to reactions. We start with a dict of roles and reactions, but we only need a list of unique reactions, so we convert the keys to a set.

```
In [3]: roles_to_reactions = PyFBA.filters.roles_to_reactions(roles)
        reactions_to_run = set()
        for role in roles_to_reactions:
            reactions_to_run.update(roles_to_reactions[role])
        print("There are {}".format(len(reactions_to_run)),
              "unique reactions associated with this genome".format(len(reactions_to_run)))
```

```
There are 1392 unique reactions associated with this genome
```

1.2.1 Read all the reactions and compounds in our database

We read all the reactions, compounds, and enzymes in the ModelSEEDDatabase into three data structures. Each one is a dictionary with a string representation of the object as the key and the PyFBA object as the value.

We modify the reactions specifically for Gram negative models (there are also options for Gram positive models, Mycobacterial models, general microbial models, and plant models).

```
In [4]: compounds, reactions, enzymes = \
            PyFBA.parse.model_seed.compounds_reactions_enzymes("gramnegative")
```

Update reactions to run, making sure that all reactions are in the list! There are some reactions that come from functional roles that do not appear in the reactions list. We're working on tracking these down, but for now we just check that all reaction IDs in *reactions_to_run* are in *reactions*, too.

```
In [5]: tempset = set()
        for r in reactions_to_run:
            if r in reactions:
                tempset.add(r)
            else:
                print("Reaction ID {}".format(r),
                      "is not in our reactions list. Skipped",
                      file=sys.stderr)
        reactions_to_run = tempset
```

```
Reaction ID rxn37218 is not in our reactions list. Skipped
```

1.2.2 Test whether these reactions grow on ArgonneLB media

We can test whether this set of reactions grows on ArgonneLB media. The media is the same one we used above, and you can download the ArgonneLB.txt and text file and put it in the same directory as this iPython notebook to run it.

(Note: we don't need to convert the media components, because the media and compounds come from the same source.)

```
In [6]: media = PyFBA.parse.read_media_file("ArgonneLB.txt")
        print("Our media has {} components".format(len(media)))
```

```
Our media has 65 components.
```

1.2.3 Define a biomass equation

The biomass equation is the part that says whether the model will grow! This is a metabolism.reaction.Reaction object.

```
In [7]: biomass_equation = PyFBA.metabolism.biomass_equation("gramnegative")
```

1.2.4 Run the FBA

With the reactions, compounds, reactions_to_run, media, and biomass model, we can test whether the model grows on this media.

```
In [8]: status, value, growth = PyFBA.fba.run_fba(compounds, reactions, reactions_to_run,
                                                   media, biomass_equation)
        print("Initial run has a biomass flux value of {} --> Growth: {}".format(value, growth))
```

```
Initial run has a biomass flux value of 4.06345328793e-14 --> Growth: False
```

1.3 Gap-fill the model

Since the model does not grow on ArgonneLB we need to gap-fill it to ensure growth. There are several ways that we can gap-fill, and we will work through them until we get growth.

As you will see, we update the *reactions_to_run list* each time, and keep the media and everything else consistent. Then we just need to run the FBA like we have done above and see if we get growth.

We also keep a copy of the original *reactions_to_run*, and a list with all the reactions that we are adding, so once we are done we can go back and bisect the reactions that are added.

```
In [9]: added_reactions = []
        original_reactions_to_run = copy.copy(reactions_to_run)
```

1.3.1 Media import reactions

We need to make sure that the cell can import everything that is in the media... otherwise it won't be able to grow. Be sure to only do this step if you are certain that the cell can grow on the media you are testing.

```
In [10]: media_reactions = PyFBA.gapfill.suggest_from_media(compounds, reactions,
                                                            reactions_to_run, media)
         added_reactions.append(("media", media_reactions))
         reactions_to_run.update(media_reactions)
```

```
In [11]: status, value, growth = PyFBA.fba.run_fba(compounds, reactions, reactions_to_run,
                                                    media, biomass_equation)
         print("The biomass reaction has a flux of {} --> Growth: {}".format(value, growth))
```

```
The biomass reaction has a flux of 1.4583713765e-15 --> Growth: False
```

1.3.2 Reactions from closely related organisms

We also gap-fill on closely related organisms. We assume that an organism is most likely to have reactions in its genome that are similar to those in closely related organisms.

```
In [12]: reactions_from_other_orgs = PyFBA.gapfill.suggest_from_roles("closest.genomes.roles",
                                                                      reactions)
         added_reactions.append(("close genomes", reactions_from_other_orgs))
         reactions_to_run.update(reactions_from_other_orgs)
```

```
In [13]: status, value, growth = PyFBA.fba.run_fba(compounds, reactions, reactions_to_run,
                                                    media, biomass_equation)
         print("The biomass reaction has a flux of {} --> Growth: {}".format(value, growth))
```

```
The biomass reaction has a flux of -3.23181490595e-14 --> Growth: False
```

1.3.3 Essential reactions

There are ~100 reactions that are in every model we have tested, and we construe these to be essential for all models, so we typically add these next!

In [14]:
```
essential_reactions = PyFBA.gapfill.suggest_essential_reactions()
added_reactions.append(("essential", essential_reactions))
reactions_to_run.update(essential_reactions)
```

In [15]:
```
status, value, growth = PyFBA.fba.run_fba(compounds, reactions, reactions_to_run,
                                          media, biomass_equation)
print("The biomass reaction has a flux of {} --> Growth: {}".format(value, growth))
```

```
The biomass reaction has a flux of 3.38622145944e-14 --> Growth: False
```

1.3.4 Subsystems

The reactions connect us to subsystems (see Overbeek et al. 2014), and this test ensures that all the subsystems are complete. We add reactions required to complete the subsystem.

In [16]:
```
subsystem_reactions = \
    PyFBA.gapfill.suggest_reactions_from_subsystems(reactions,
                                                    reactions_to_run,
                                                    threshold=0.5)
added_reactions.append(("subsystems", subsystem_reactions))
reactions_to_run.update(subsystem_reactions)
```

In [17]:
```
status, value, growth = PyFBA.fba.run_fba(compounds, reactions, reactions_to_run,
                                          media, biomass_equation)
print("The biomass reaction has a flux of {} --> Growth: {}".format(value, growth))
```

```
The biomass reaction has a flux of 4.13676770622e-13 --> Growth: False
```

1.3.5 Orphan compounds

Orphan compounds are those compounds which are only associated with one reaction. They are either produced, or trying to be consumed. We need to add reaction(s) that complete the network of those compounds.

You can change the maximum number of reactions that a compound is in to be considered an orphan (try increasing it to 2 or 3).

In [18]:
```
orphan_reactions = PyFBA.gapfill.suggest_by_compound(compounds, reactions,
                                                     reactions_to_run,
                                                     max_reactions=1)
added_reactions.append(("orphans", orphan_reactions))
reactions_to_run.update(orphan_reactions)
```

In [19]:
```
status, value, growth = PyFBA.fba.run_fba(compounds, reactions, reactions_to_run,
                                          media, biomass_equation)
print("The biomass reaction has a flux of {} --> Growth: {}".format(value, growth))
```

```
The biomass reaction has a flux of 1000.0 --> Growth: True
```

1.4 Trimming the model

Now that the model has been shown to grow on ArgonneLB media after several gap-fill iterations, we should trim down the reactions to only the required reactions necessary to observe growth.

In [20]:
```
reqd_additional = set()

# Begin loop through all gap-filled reactions
while added_reactions:
    ori = copy.copy(original_reactions_to_run)
    ori.update(reqd_additional)
    # Test next set of gap-filled reactions
    # Each set is based on a method described above
    how, new = added_reactions.pop()

    # Get all the other gap-filled reactions we need to add
    for tple in added_reactions:
        ori.update(tple[1])

    # Use minimization function to determine the minimal
    # set of gap-filled reactions from the current method
    new_essential = PyFBA.gapfill.minimize_additional_reactions(ori, new, compounds,
                                                                reactions, media,
                                                                biomass_equation)
    # Record the method used to determine
    # how the reaction was gap-filled
    for new_r in new_essential:
        reactions[new_r].is_gapfilled = True
        reactions[new_r].gapfill_method = how
    reqd_additional.update(new_essential)

# Combine old and new reactions
all_reactions = original_reactions_to_run.union(reqd_additional)
```

```
At the beginning the base list has 2037  and the optional list has 3179 reactions
At the beginning the base list has 1796  and the optional list has 227 reactions
At the beginning the base list has 1751  and the optional list has 109 reactions
At the beginning the base list has 1457  and the optional list has 247 reactions
The set of 'base' reactions results in growth so we don't need to bisect the optional set
```

In [21]:
```
status, value, growth = PyFBA.fba.run_fba(compounds, reactions, all_reactions,
                                          media, biomass_equation)
print("The biomass reaction has a flux of {} --> Growth: {}".format(value, growth))
```

```
The biomass reaction has a flux of 266.346949801 --> Growth: True
```

1.5 Other gap-filling techniques

Besides those methods we have described above, listed here are other methods that can be used to gap-fill your model. This list will continue to grow over time as we create new techniques to identify reactions and compounds that should be added to your model.

1.5.1 Probable reactions

Probable reactions are those reactions whose probability is based on whether there is a protein associated with the reaction and if the reaction's compounds are currently present in the model. Above a certain probability threshold, those reactions will be added to the model.

```
In [22]: probable_reactions = PyFBA.gapfill.compound_probability(reactions, reactions_to_run,
                                                                  cutoff=0, rxn_with_protiens=True)
```
