# Supplementary material for: From DNA to FBA: How to Build Your Own Genome-Scale Metabolic Model
Source: Front Microbiol. 2016 Jun 17;7:907. doi: 10.3389/fmicb.2016.00907 (PMC4911401; doi:10.3389/fmicb.2016.00907)
Supplement: Supplementary file 6 [file Data_Sheet_5.PDF]

Supplemental Material
PyFBA

Using an SBML model

Daniel A. Cuevas *Robert A. Edwards*
San Diego State University

1 Using an SBML model

1.1 Getting started

1.1.1 Installing libraries

Before you start, you will need to install a couple of libraries:

The ModelSeedDatabase has all the biochemistry we'll need. You can install that with `git clone`.

The PyFBA library has detailed installation instructions. Don't be scared, its mostly just `pip install`.

(Optional) Also, get the SEED Servers as you can get a lot of information from them. You can install the git python repo from github. Make sure that the SEED_Servers_Python is in your PYTHONPATH.

We start with importing some modules that we are going to use.

We import *sys* so that we can use standard out and standard error if we have some error messages. We import *copy* so that we can make a deep copy of data structures for later comparisons. We import *print_function* so that we can use the print using Python 3.X syntax. Then we import the *PyFBA* module to get started.

```
In [1]: import sys
        import copy
        import PyFBA
        from __future__ import print_function
```

1.2 Running an SBML model

If you have run your genome through RAST, you can download the SBML model and use that directly.

We have provided an SBML model of *Citrobacter sedlakii* that you can download and use. You can right-ctrl click on this link and save the SBML file in the same location you are running this iPython notebook.

We use this SBML model to demonstrate the key points of the FBA approach: defining the reactions, including the boundary, or drainflux, reactions; the compounds, including the drain compounds; the media; and the reaction bounds.

We'll take it step by step!

We start by parsing the model:

```
In [2]: sbml = PyFBA.parse.parse_sbml_file("Citrobacter_sedlakii.sbml")
```

1.2.1 Find all the reactions and identify those that are boundary reactions

We need a set of reactions to run in the model. In this case, we are going to run all the reactions in our SBML file. However, you can change this set if you want to knock out reactions, add reactions, or generally modify the model. We store those in the `reactions_to_run` set.

The boundary reactions refer to compounds that are secreted but then need to be removed from the reactions_to_run set. We usually include a consumption of those compounds that is open ended, as if they are draining away. We store those reactions in the uptake_secretion_reactions dictionary.

```
In [3]: # Get a dict of reactions.
        # The key is the reaction ID, and the value is a metabolism.reaction.Reaction object
        reactions = sbml.reactions
        reactions_to_run = set()
        uptake_secretion_reactions = {}
        biomass_equation = None
        for r in reactions:
            if 'biomass_equation' in reactions[r].name.lower():
                biomass_equation = reactions[r]
                continue
            is_boundary = False
            for c in reactions[r].all_compounds():
                if c.uptake_secretion:
                    is_boundary = True
            if is_boundary:
                reactions[r].is_uptake_secretion = True
                uptake_secretion_reactions[r] = reactions[r]
            else:
                reactions_to_run.add(r)
```

At this point, we can take a look at how many reactions are in the model, not counting the biomass reaction:

```
In [4]: print("There are {} reactions in the model".format(len(reactions)))
        print("There are {}".format(len(uptake_secretion_reactions)),
              "uptake/secretion reactions in the model")
        print("There are {}" "reactions to be run in the model")
```

```
There are 1574 reactions in the model
There are 174 uptake/secretion reactions in the model
There are 1399 reactions to be run in the model
```

1.2.2 Find all the compounds in the model, and filter out those that are secreted

We need to filter out uptake and secretion compounds from our list of all compounds before we can make a stoichiometric matrix.

```
In [5]: # Get a dict of compounds.
        # The key is the string representation of the compound and
        # the value is a metabolite.compound.Compound object
        all_compounds = sbml.compounds
        # Filter for compounds that are boundary compounds
        filtered_compounds = {}
        for c in all_compounds:
            if not all_compounds[c].uptake_secretion:
                filtered_compounds[c] = all_compounds[c]
```

Again, we can see how many compounds there are in the model.

```
In [6]: print("There are {} total compounds in the model".format(len(all_compounds)))
        print("There are {} compounds that are not involved in uptake and secretion".format(len(filtere
```

```
There are 1475 total compounds in the model
There are 1301 compounds that are not involved in uptake and secretion
```

And now we have the size of our stoichiometric matrix! Notice that the stoichiometric matrix is composed of the reactions that we are going to run and the compounds that are in those reactions (but not the uptake/secretion reactions and compounds).

```
In [7]: print("The stoichiometric matrix will be {} reactions by {} compounds".format(len(reactions_to_
                                                                len(filtered_compounds)))
```

```
The stoichiometric matrix will be 1399 reactions by 1301 compounds
```

1.2.3 Read the media file, and correct the media names

In our media directory, we have a lot of different media formulations, most of which we use with the Genotype-Phenotype project. For this example, we are going to use Lysogeny Broth (LB). There are many different formulations of LB, but we have included the recipe created by the folks at Argonne so that it is comparable with their analysis. You can download ArgonneLB.txt and put it in the same directory as this iPython notebook to run it.

Once we have read the file we need to correct the names in the compounds. Sometimes when compound names are exported to the SBML file they are modified slightly. This just corrects those names.

```
In [8]: # Read the media file
        media = PyFBA.parse.read_media_file('ArgonneLB.txt')
        # Correct the names
        media = PyFBA.parse.correct_media_names(media, all_compounds)
```

1.2.4 Set the reaction bounds for uptake/secretion compounds

The uptake and secretion compounds typically have reaction bounds that allow them to be consumed (i.e. diffuse away from the cell) but not produced. However, our media components can also increase in concentration (i.e. diffuse to the cell) and thus the bounds are set higher. Whenever you change the growth media, you also need to adjust the reaction bounds to ensure that the media can be consumed!

```
In [9]: # Adjust the lower bounds of uptake secretion reactions
        # for things that are not in the media
        for u in uptake_secretion_reactions:
            is_media_component = False
            for c in uptake_secretion_reactions[u].all_compounds():
                if c in media:
                    is_media_component = True
            if not is_media_component:
                reactions[u].lower_bound = 0.0
                uptake_secretion_reactions[u].lower_bound = 0.0
```

1.2.5 Run the FBA

Now that we have constructed our model, we can run the FBA!

```
In [10]: status, value, growth = PyFBA.fba.run_fba(filtered_compounds, reactions,
                                                   reactions_to_run, media, biomass_equation,
                                                   uptake_secretion_reactions)
         print("The FBA completed with a flux value of {} --> growth: {}".format(value, growth))
```

```
The FBA completed with a flux value of 281.841757437 --> growth: True
```
